# Incidence and predictors of mortality among neonates with congenital heart disease in Ethiopia: a retrospective cohort study

**DOI:** 10.1186/s12887-024-05023-3

**Published:** 2024-08-31

**Authors:** Abatwoy Ayfokru, Sisay Shewasinad, Fuad Ahmed, Mitku Tefera, Genet Nigussie, Emawaysh Getaneh, Leweyehu Alemaw Mengstie, Wegayehu Zeneb Teklehaimanot, Worku Abemie Seyoum, Mohammed Tessema Gebeyehu, Metages Alemnew, Bekahegn Girma

**Affiliations:** 1 Department of Pediatrics and Child Health Nursing, School of Nursing and Midwifery, Asrat Woldeyes Health Sciences campus, Debre Berhan University, Debre Berhan, Ethiopia; 2 Department of Midwifery, Debre Berhan Health Science College, Debre Berhan, Ethiopia; 3 Department of Nursing, Mida-Woremo Primary Hospital, Amhara region, Ethiopia; 4 Department of Midwifery, School of Nursing and Midwifery, Asrat Woldeyes Health Sciences campus, Debre Berhan University, Debre Berhan, Ethiopia; 5 School of medicine, Asrat Woldeyes Health Sciences campus, Debre Berhan University, Debre Berhan, Ethiopia

**Keywords:** Congenital heart disease, Neonate, Mortality, Incidence, Predicators, Ethiopia

## Abstract

**Background:**

Neonatal mortality poses a significant public health challenge in sub-Saharan Africa, with congenital heart disease emerging as the leading cause of morbidity and mortality among neonates, especially in countries like Ethiopia. Despite efforts to reduce neonatal mortality rates, Ethiopia continues to experience an increased mortality rate, particularly among neonates with congenital heart disease. This study aims to investigate the incidence and predictors of mortality in this vulnerable population within Ethiopia.

**Method:**

A retrospective cohort study was conducted at an institution, involving 583 randomly selected neonates diagnosed with congenital heart disease. In the current study, the dependent variable was survival status. Data entry utilized EpiData data version 4.6, and analysis was performed using STATA version 16. Probability of death was compared using the log-rank test and Kaplan-Meier failure curve. Significant predictors were identified using bivariable and multivariate Cox regression. Model fitness and proportional hazard assumptions were evaluated using the Cox-Snell graph and Global test, respectively. Associations were assessed by adjusted hazard ratios with 95% confidence intervals.

**Results:**

The study participants were followed for 4844 days. The mortality rate was 9.9%. The incidence density was 11.9 per 1000 person-days of observation. Neonatal sepsis (AHR: 2.24; 95% CI [1.18–4.23]), cyanotic congenital heart disease (AHR: 3.49; 95% CI [1.93–6.28]), home delivery (AHR: 1.9; 95% CI [1.06–3.6]), maternal history of gestational diabetes mellitus (AHR: 1.94; 95% CI [1.04–3.61]), and having additional congenital malformations (AHR: 2.49; 95% CI [1.33–4.67]) were significant predictors for neonatal mortality.

**Conclusion and recommendation:**

The incidence density of mortality was high compared to studies conducted in developed countries. Neonatal sepsis, type of congenital heart disease, place of delivery, maternal history of gestational diabetes mellitus, and having an additional congenital malformation were significant predictors of mortality among neonates with congenital heart disease. Therefore, healthcare providers should pay special attention to patients with identified predictors. Furthermore, the Federal Ministry of Health, stakeholders, and policymakers should collaborate to address this issue.

**Supplementary Information:**

The online version contains supplementary material available at 10.1186/s12887-024-05023-3.

## Background

Congenital heart diseases (CHDs) involve major structural abnormalities in the heart or intrathoracic great vessels due to congenital anomalies, which disrupt blood flow through the heart and body [1, 2]. CHDs represent a prevalent category of congenital defects, constituting approximately one-third of all major congenital anomalies [3]. It has two types a cyanotic and cyanotic [4, 5].

Approximately 30–40% of neonates with congenital heart disease (CHD) exhibit symptoms during their first year of life, with 60% of these cases being diagnosed within the first month after birth [6]. The mortality rate of newborns diagnosed with congenital heart disease (CHD) continues to be a pressing global issue. Despite significant progress in medical treatments, newborns with CHD still confront heightened risks of mortality due to the intricate nature of their cardiac abnormalities. Globally, CHD contributes to around 25% of all neonatal deaths related to congenital anomalies, and its incidence is increasing, underscoring a considerable gap in diagnostic capabilities, especially in Africa [1].

As a result, CHD significantly affects the daily routines and financial stability of family members, leading to a decline in their quality of life. Moreover, neonates with heart disease often experience challenges such as malnutrition, infections, and pulmonary hypertension, particularly in low-income countries like Ethiopia, complicating the treatment process further. Altogether, caring for these neonates presents a formidable challenge [7–10].

Globally, over the last 15 years 1.35 million live births are diagnosed with CHD every year [5]. Majority were born in locations that included Africa with little or no care. Due to this, 90% of newborns with CHD received inadequate medical care [11] and 30% of neonates with CHD die in the neonatal period [3, 12, 13].

In Africa, where only 20 cardiac centers found, the incidence and mortality of neonates with CHD is increased [14, 15] and underreported [11, 14]. In Ethiopia, where the magnitude of neonatal mortality is increased in recent years there is one cardiac center which served for 56 million people [9]. Due to this and other factors the mortality of neonates with CHD in Ethiopia is still unknown [16, 17].

The country has instituted various services such as preconception, essential newborn, antenatal, and postnatal care to alleviate the burden of mortality [18, 19]. Nevertheless, mortality rates remain high, and there is insufficient evidence regarding the incidence and predictors of mortality among neonates with CHD in Ethiopia. Consequently, this study aimed to explore the incidence and predictors of mortality among neonates with CHD in Ethiopia.

## Method

### Study area and period

This study was conducted at Amhara regional state of Ethiopia. As indicated by the 2020 Health and Health Related Indicators report published by the Ministry of Health (MoH), Amhara region is a home to 82 hospitals, 861 health centers, and 3,565 health posts. Among these hospitals, the University of Gondar, Dessie, Felege-Hiwot, Tibebe-Ghion, Debre Markos, Woldia, Debre Tabor, and Debre Berhan are classified as Comprehensive Specialized Hospitals. The current study was conducted in the above specialized comprehensive hospitals which provide cardiac service.

### Study design

Institutional based retrospective cohort study was conducted.

### Source population

The source population included all neonates diagnosed with congenital heart disease admitted to the neonatal intensive care units (NICUs) of Comprehensive Specialized Hospitals in the Amhara region.

### Study population

Neonates diagnosed with congenital heart disease were specifically chosen for admission to the NICU wards of selected Comprehensive Specialized Hospitals in the Amhara regional state.

### Eligibility criteria

All newly diagnosed and admitted neonates with congenital heart disease from January 1^st^ 2018 to December 30^th^ 2022 were included. Infants lacking complete records of the outcome variable were excluded from consideration.

### Sample size determination

The sample size required for this study was determined by STATA version 16 (Cox model), based on the following assumptions: a hazard ratio (HR) of 1.4 for the selected covariate of interest (maternal educational status) and a probability of failure (death) of 0.186 from study done in Ethiopia [20], a standard deviation (SD) of 0.4, a margin of error of 5%, and a confidence interval (CI) of 95% to achieve 80% power. Accounting for a 10% non-response rate, the final sample size required for this study was determined to be 587.

$$\begin{array}{cc}\mathrm n=\frac{\mathrm E\left(\mathrm{\alpha,\beta,\psi}\right)}{\mathrm{PE}\left(\mathrm S\left(\mathrm t\right),\mathrm L\left(\mathrm t\right),\mathrm R,\mathrm L\right)}&\mathrm E=\frac{\left(\mathrm Z1-\mathrm\alpha2+\mathrm Z1-\mathrm\beta\right)2}{\mathrm{Log}\left(\mathrm{HR}\right)2\mathrm q1\mathrm q0}\end{array}$$

### Sampling technique and sampling procedure

**Fig. 1:**
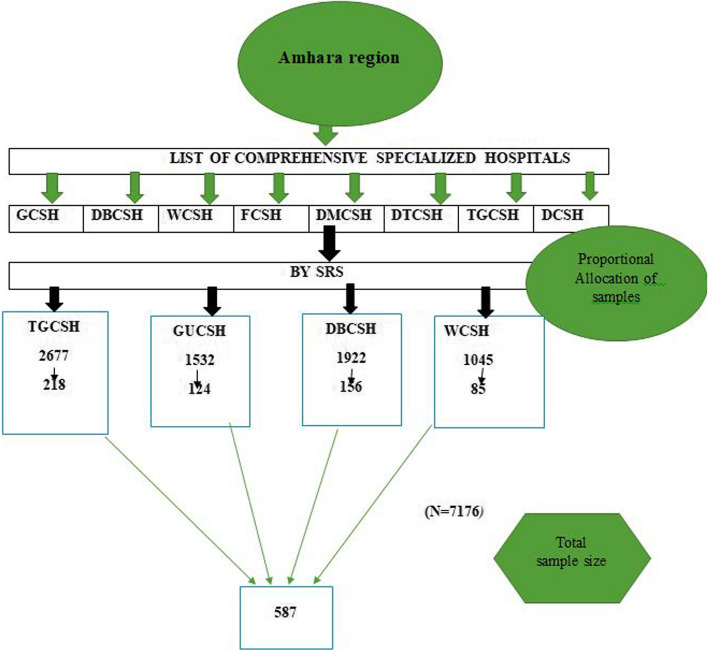
Schematic presentation of the sampling for incidences and predictor of mortality among neonates with CHD in Ethiopia, 2024

Four specialized hospitals were randomly selected using a lottery method. Study participants were chosen through systematic random sampling, where the sampling interval for each hospital was determined by dividing the number of eligible participants by the allocated sample size. Initial participants were selected using a systematic approach at every 12th interval (calculated as 7176/587 = 12). The first participants were chosen by lottery, and subsequent selections occurred every 12th interval until the desired sample size was achieved (Fig. 1).

### Data collection tool and procedure

Data from NICU registration books were collected using a pretested, pre-structured checklist. This checklist encompassed socio-demographic, maternal behavioral and medical, obstetric, and neonatal variables. The retrospective follow-up period was determined by the time from CHD admission to discharge. Data recordings were included based on eligibility criteria. The questionnaire, adapted from literature and guidelines [21–29], was prepared in English. The data were collected by healthcare professionals stationed at treatment centers.

### Operational definitions

In this study, the date of admission served as the starting point for follow-up, which extended until the infant reached 28 days of life.

#### Event

death of neonates with congenital heart disease while in the hospital.

#### Censored

neonates with congenital heart disease who were improved, discharged, or against medical treatment, and referred to others center.

#### Time scale

days.

#### Co-morbidities

Newborns with CHD and have other illnesses such as sepsis and under nutrition [30].

#### Congenital heart defect

Major or minor congenital anomalies defined as anatomical structural and functional defect present at birth which was confirmed by pediatricians or echocardiography [31].

### Data quality control

The checklist was pre-tested with 5% of participants outside the study area to ensure data quality. It was then standardized and validated with input from clinical and academic experts. The study’s tool was validated and confirmed reliable.

Data collectors and supervisors, senior BSc nurses with NICU experience, were chosen for each hospital. They underwent one-day training on the study’s objectives, data collection tools, methods, and ethical considerations. Supervisors oversaw the data collection, and spot-checks were conducted by the principal investigator and supervisors to ensure completeness and consistency of the collected data.

### Data processing and analysis

After ensuring data completeness and consistency, they were coded and entered into EpiData version 4.6, then exported to STATA version 16 for cleaning and analysis, using summary statistics, text, graphs, and tables.

Multicollinearity was assessed using variance inflation factors. Descriptive statistics, Kaplan-Meier graphs, and log-rank tests were performed. The proportional hazard assumption was tested using Schoenfeld residuals (global test), and model fitness was evaluated with a Cox-Snell residual test. Bivariable and multivariate Cox proportional hazard regression models identified predictors, presenting results with Adjusted Hazard Ratios (AHR) and 95% Confidence Intervals (CI).

## Results

A total of 587 neonate’s charts were scrutinized, of which 583 were eligible and complete with a response rate of 99.32%.

### Socio-demographic characteristics of mother of neonate

In this study, 310 neonates (53.17%) had mothers aged over 35 years, and 506 (86.79%) were born to married mothers. Furthermore, 404 participants (69.3%) were over seven days old, and 393 (67.41%) were female. Additionally, 418 (83.53%) of the study participants lived in rural areas (Table 1).

**Table 1:** Socio-demographic related characteristics for mortality of neonates with congenital heart disease in Ethiopia, 2024 (*n* = 583)

Characteristics	Category	Survival status		Frequency (%)
		Censored	Death	
Maternal age	<= 20 Year	31	6	37 (6.35)
	20 = 34 Year	215	21	236 (40.48)
	>=35 Year	274	36	310 (53.17)
Sex of neonates	Male	168	22	190 (32.59)
	Female	357	36	393 (67.41)
Age of neonates	<=7 day	146	33	179 (30.70)
	> 7 day	379	25	404 (69.30)
Marital Status	Married	459	47	506 (86.79)
	Single	9	4	13 (2.23)
	Divorced	34	7	41 (7.03)
	Widowed	15	7	23 (3.95)
Residency	Urban	77	19	96 (16.47)
	Rural	448	39	487 (83.53)

### Maternal behavioral, medical and obstetric related factors

Among the neonates, 18.18% were born to mothers who consumed alcohol during pregnancy, and 20.24% were born to mothers who chewed khat. 13.35% had mothers with gestational diabetes mellitus, and 6.66% had mothers with cardiac problems. Furthermore, 9.78% were born to HIV-infected mothers, and 22.13% had a family history of congenital heart disease (Table 2).

**Table 2:** Maternal behavioral and medical related characteristics for mortality of neonates with congenital heart disease in Ethiopia, 2024 (*n* = 583)

Characteristics	Category	Survival Status		Frequency (%)
		Censored	Death	
History of Alcohol intake during Pregnancy	Yes	85	21	106 (18.18)
	No	440	37	477 (81.82)
History of Smoking intake during this pregnancy	Yes	127	8	135 (23.16)
	No	398	50	448 (76.84)
History of Chewing chat during this pregnancy	Yes	114	4	118 (20.24)
	No	411	54	465 (79.76)
GDM	Yes	52	20	72 (13.35)
	No	473	38	511 (87.65)
Hypertensions	Yes	11	6	17 (2.92)
	No	509	57	566 (97.08)
Cardiac problems	Yes	34	6	40 (6.86)
	No	491	52	543 (93.14)
Syphilis	Yes	30	7	37 (6.35)
	No	495	51	546 (93.65)
HIV/AIDS	Yes	37	20	57 (9.78)
	No	488	38	526 (90.22)
TB	Yes	17	5	22 (3.77)
	No	508	53	561 (96.23)
Anemia	Yes	33	5	38 (6.52)
	No	492	53	545 (93.48)
History of CHD	Yes	110	19	129 (22.13)
	No	415	39	454 (77.87)

Most of the mothers of the neonates (506, 86.79%) had two or more pregnancies, 452 (77.53%) experienced spontaneous vaginal births, and 301 (51.63%) had received antenatal care (ANC) follow-up. Additionally, 108 neonates (18.52%) were born to mothers who had a history of neonatal death. Lastly, 165 neonates (28.30%) were born at home (Table 3).

**Table 3:** Obstetric related characteristics for mortality of neonates with congenital heart disease in Ethiopia, 2024 (*n* = 583)

Characteristics	Category	Survival status		Frequency (%)
		Censored	Death	
ANC follow Up visits	Yes	266	35	301 (51.63)
	No	259	23	282 (48.34)
Ferrous sulphate with folic acid provided	Yes	283	22	305 (52.35)
	No	242	36	278 (47.68)
Obstetric *U/S performed within 24 WK of GA*	Yes	116	13	129 (22.13)
	No	409	45	454 (77.87)
Gravidity	Prim gravid	70	7	77 (13.21)
	Multi gravid	455	51	506 (86.79)
History of Abortion	Yes	89	17	106 (18.18)
	No	436	41	477 (81.82)
History of neonatal death	Yes	90	18	108 (18.52)
	No	435	40	475 (81.48)
History of APH	Yes	36	7	43 (7.38)
	No	489	51	540 (92.62)
History of prolonged labour	Yes	38	6	44 (7.55)
	No	487	52	539 (92.45)
History of Premature Rapture of membranes	Yes	32	12	44 (7.55)
	No	493	46	539 (92.45)
History of RH Factors	Yes	49	6	55 (9.43)
	No	476	52	528 (90.57)
Types of birth	Single	252	18	270 (46.31)
	multiple	273	40	313 (53.69)
Mode of delivery	SVD	409	43	452 (77.53)
	C/S	116	15	131 (22.47)
Place of delivery	Home	133	32	165 (28.30)
	Health center	148	7	155 (26.59)
	Hospital	244	19	263 (45.11)

### Neonatal related characteristics

Among the newborns with CHD in the NICU, 389 (66.72%) had low birth weight, 260 (44.60%) had neonatal sepsis, 172 (29.50%) had PNA, 320 (54.89%) were born prematurely, and 49 (8.10%) had another cardiac anomaly. The mean birth weight at admission was 2253 g (Table 4).

**Table 4:** Neonatal related characteristics for mortality of neonates with CHD in Ethiopia, 2024 (*n* = 583)

Characteristics	Category	Survival status		Frequency (%)
		Censored	Death	
Gestational age	<=37 WK	285	35	320 (54.89)
	>=37WK	240	23	263 (45.11)
5th minutes Apgar score Status	Normal	32	8	40 (6.86)
	Moderate	296	17	313 (53.69)
	Severs	190	40	230 (39.45)
Birth weight	<=2500	351	38	389 (66.72)
	>=2500	174	20	194 (33.28)
Time of Diagnosis to CHD	Early diagnosis	192	25	217 (37.22)
	Late diagnosis	333	33	366 (62.78)
Types of CHD	Cyanotic CHD	84	33	117 (20.07)
	A cyanotic CHD	441	25	466 (79.93)
Others congenital malformation	Yes	35	14	49 (8.40)
	No	490	44	534 (91.60)
Comorbidity	Yes	250	41	291 (49.91)
	No	275	17	292 (50.09)
Types of intervention to CHD	Medical	459	55	514 (88.16)
	Surgical	64	5	69 (11.84)
Air way Resuscitation needed during delivery	Yes	323	51	374 (64.15)
	No	202	7	209 (35.85)
Perinatal asphyxia	Yes	165	7	172 (29.50)
	No	360	51	411 (70.50)
Sepsis	Yes	217	43	260 (44.60)
	No	308	15	323 (55.40)
Severe acute malnutrition	Yes	108	25	133 (22.81)
	No	417	33	450 (77.19)

### Overall survival function and probability

The overall mean survival time of neonates admitted to NICU in the study was 8 days with 90.3% (95% CI 0.86–0.93) a SD ± 0.015. This study also showed that the probability of neonatal survival at the 7th and 14th day of hospital stay was 92.5% (95% CI 89 94) SD ± = 0.013) and 82.1% (95% CI: 76–86) SD ± = 0.025), respectively. At the 20 days of hospital stay, the overall survival probability of neonates was 74.1% (95% CI: 65–80) with a standard error of 0.039 (Annex 1).

**Fig. 2:**
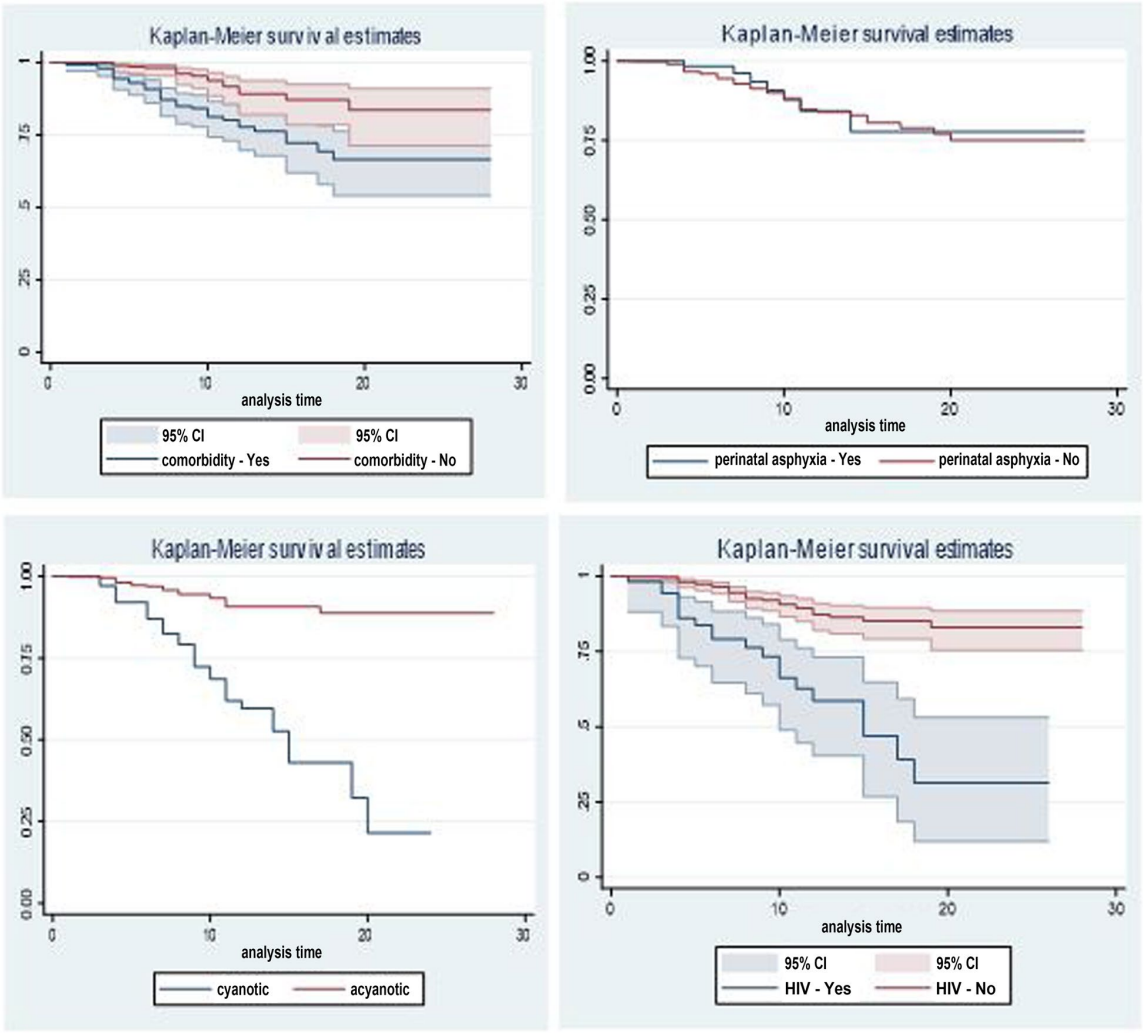
Kaplan meier graph for selected predictors of mortality to neoates with CHD in Ethiopia, 2024

 To assess the survival difference between selected covariates Kaplan Meier graph and log rank test were done. According to both Kaplan Meier graph and log rank test there is survival difference between neonates who had comorbidity and hadn’t; neonates who had comorbidity had low survival rate (Log rank test; Pr > chi2 = 0.0001). Moreover, neonates who had a cyanotic CHD had less survival rate. However, there was no survival difference between neonates (log rank test result; Pr > chi^2^ = 0.00) (Fig. 2).

### Survival status of neonates with CHD

**Fig. 3:**
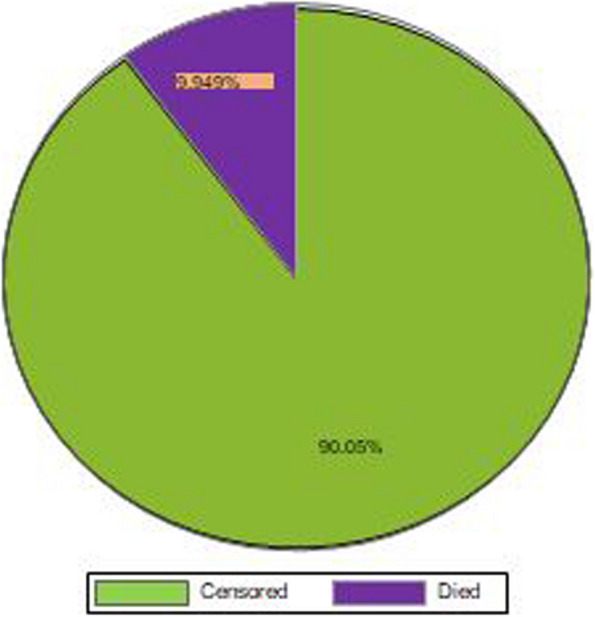
Survival status of neonates with CHD in Ethiopia, 2024 (*n* = 583)

**Fig. 4:**
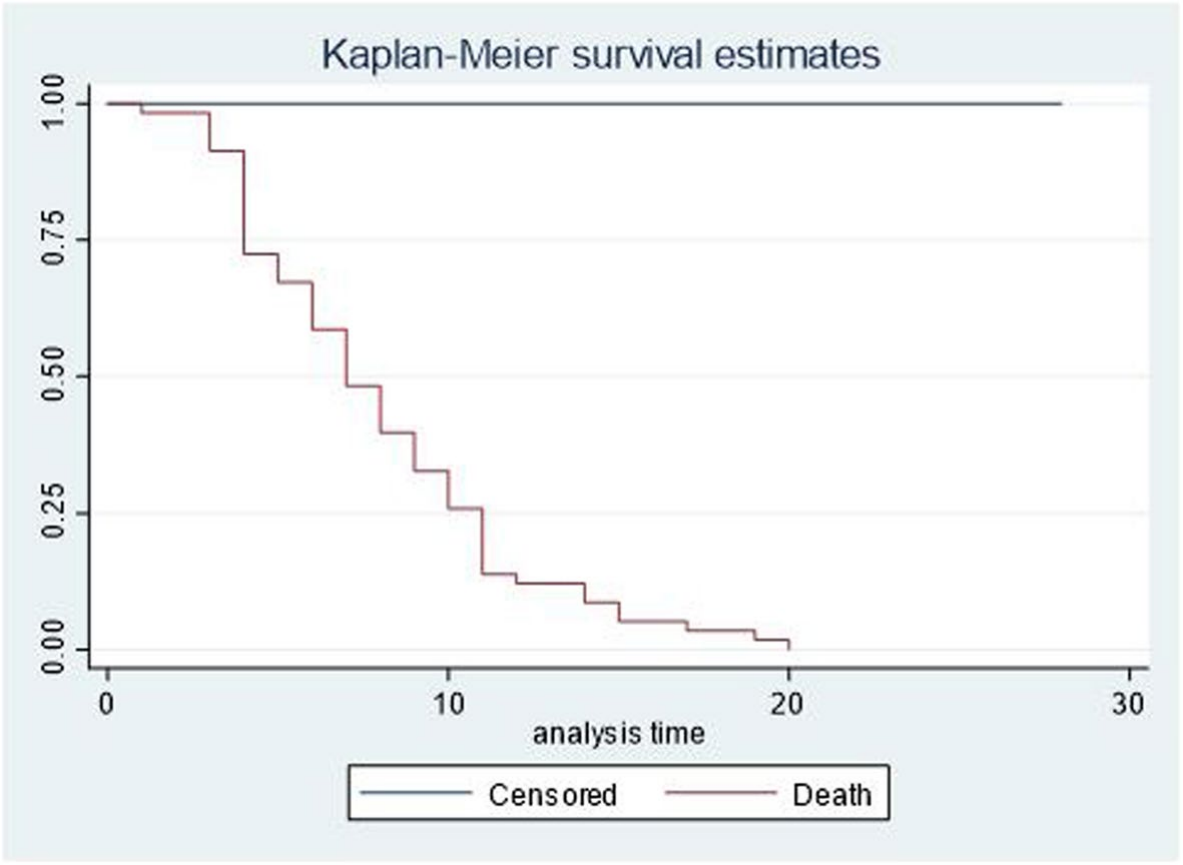
Median survival time for neonates with CHD in Ethiopia, 2024

A total of 583 neonates with CHD were followed from admission to 28 days with 4844 person day observations. In the current study, 58 (9.79%) of neonates died and 525 (90.05%) of them censored (Fig. 3). The median survival time was at 7 days (Fig. 4). The overall incidence density rate of mortality was 11.9 [95% CI (9.00–15.00)] per 1000 person days observation. The incidence density rate of mortality among cyanotic CHD neonates and a cyanotic CHD was 37.3 and 6.3 per 1000 person day observations respectively.

**Fig. 5:**
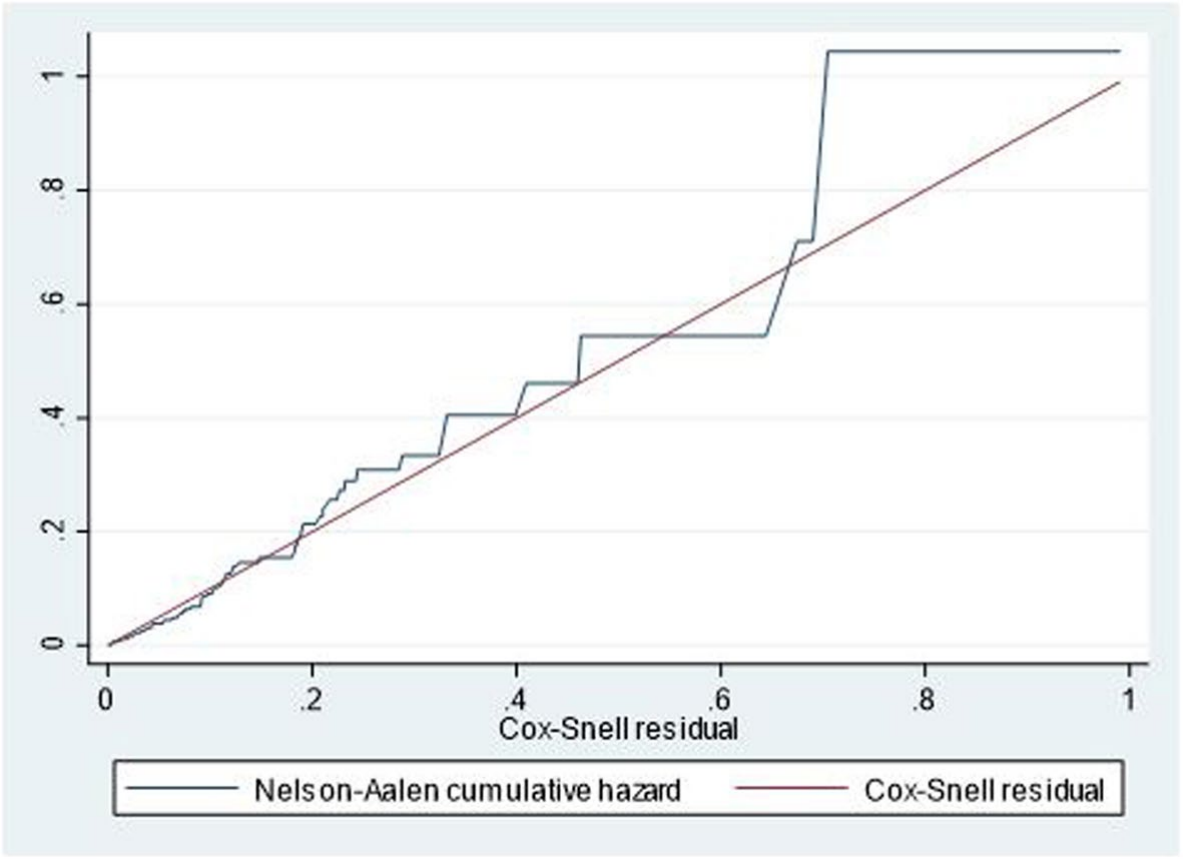
Cox-Snell residual cumulative hazard graph for neonates with CHD in Ethiopia, 2024

### Predictors of mortality for neonates with CHD

Before conducting the analysis, multicollinearity, model fitness, and proportional hazard assumption were assessed. The model was fitted and verified using the Cox-Snell graph (Fig. 5), confirming that the assumption was not violated (with a global test value of 0.6337). Additionally, the Variance Inflation Factor (VIF) was found to be 1.06.

In the bivariable Cox regression model, ten variables were considered: gestational diabetic mellitus (GDM), place of delivery, presence of other congenital malformation, sepsis, need for resuscitation during delivery, type of CHD, maternal HIV status, maternal alcohol intake, history of neonatal death, and premature rupture of membrane. These variables were eligible and selected for multivariate analysis. However, only five of them place of delivery, sepsis, type of CHD, presence of other congenital malformations, and GDM remained significant predictors for mortality among neonates with CHD.

Neonates diagnosed with sepsis had a hazard 2.24 times greater than their counterparts [Adjusted Hazard Ratio (AHR): 2.24; 95% CI (1.18–4.23)]. Those with cyanotic congenital heart disease faced a 3.5 times higher risk of mortality [AHR: 3.49; 95% CI (1.93–6.28)] compared to those with non-cyanotic CHD.

Additionally, neonates born at home exhibited a 1.9 times higher hazard of death [AHR: 1.9; 95% CI (1.06–3.6)] than those delivered in a healthcare institution. Neonates with a maternal history of GDM had a mortality rate 1.9 times higher [AHR: 1.94; 95% CI (1.04–3.61)] than their counterparts. Lastly, neonates with other congenital malformations were 2.5 times more likely to die [AHR: 2.49; 95% CI (1.33–4.67)] than those without such malformations (Table 5).

**Table 5:** Bivariable and multivariate analysis result for predictors of mortality to neonates with CHD in Ethiopia, 2024 (*n* = 583)

Characteristics	Category	CHR (95%CI)	AHR (95% CI)
Alcohol	Yes	1	
	No	0.40 (0.23–0.69)	0.55 (0.29 − 1.04)
HIV/AIDS	Yes	1	
	No	0.21 (0.12–0.36)	0.67 (0.35–1.28)
Gestational diabetic mellitus	No	1	
	yes	0.24(0.14-0 0.41)	1.94 (1.04–3.61)*
Premature Rapture of membrane	Yes		
	No	0.48 (0.27–0.84)	1.03 (0.47–2.27)
History of Neonatal death	Yes	1	
	No	0.37 (0.19–0.70)	0.82 (0.42–1.60)
Types of CHD	A cyanotic	1	
	Cyanotic	5.76 (3.42–9.70)	3.49 (1.93–6.28)*
Others congenital malformation	No	1	
	Yes	0.25 (0.13–0.46)	2.49 (1.33–4.67)*
History of resuscitation Needed during delivery	No	1	
	Yes	0.28 (0.12–0.61)	1.43 (0.76–2.88)
Place of delivery	Health center	1	
	Home	4.27 (1.88–9.69)	1.9 (1.06–3.60)*
Sepsis	No	1	
	Yes	4.16(2.31–7.49)	2.24 (1.18–4.23)*

*significant predictors for mortality of neonates with CHD

## Discussion

The aim of this study was to assess the incidence and predictors of mortality among neonates with CHD in Ethiopia. The mortality rate among neonates with CHD was 9.79%. The mortality incidence among neonates with congenital heart disease was 11.9 [95% CI (0.009–0.015)] per 1000 person-days of observation. Additionally, the median survival time was 7 days.

The Mortality rate revealed in this study was low as compared to studies conducted in Developing countries [23]. However, the rate was high as compared to studies conducted in Taiwan [32]. This variability could be attributed to differences in the quality and availability of cardiac services, as well as disparities in population characteristics. It’s worth noting that the study conducted in developing countries focused solely on critical neonates with CHD.

The incidence reported in this study was in line with studies conducted in China [33, 34], Norway [35] and Turkey [36]. However, it is was higher as compared to studies conducted in Europe [37], Spain [38], Sri lanka [39], united states of America (USA) [40], France [41] and Brazil [42]. Lastly, it low as compared to study done globally [43] and Brazil [44]. The potential explanation might stem from considerable disparities in socioeconomic status, study settings, and the presence of specialized cardiac surgeons and facilities. Furthermore, it could be attributed to variances in neonatal intensive care unit conditions and maternal healthcare service utilization.

Neonates who had neonatal sepsis had more risk for mortality. This finding was in line with studies conducted in Ethiopia, [27, 45–47], Indonesia [48] and USA [49]. This might be due to the distinct cardiovascular pathophysiology observed in children with congenital heart disease (CHD) compared to their peers without CHD, the standard diagnosis and management protocols for sepsis cannot be applied directly to this population which underestimates the effect of sepsis.

Neonates with cyanotic congenital heart disease had higher mortality rate. This finding was consistent with a study done in Georgia (69) and Spain (59). This similarity arises from the fact that infants with CHD have critical early difficulties that necessitate emergency care because their aorta pumps mixed blood, which raises the chance of mortality.

Home delivered neonates with CHD had more hazard of mortality. This finding was supported by studies conducted in sub-Sahara Africa [50] and Ethiopia [51]. This could be because newborns delivered at home may not receive immediate resuscitation, thereby increasing the risk of birth complications.

Neonates born to mothers with a history of maternal gestational diabetes mellitus (GDM) exhibited a heightened risk of mortality. This study’s discovery aligns with research conducted in Ethiopia [51]. This correlation may stem from GDM leading to hypoglycemia at birth, respiratory distress, polycythemia, elevated bilirubin levels, and an increased likelihood of preterm birth [52].

Finally, neonates with congenital heart disease (CHD) who also presented with additional congenital malformations faced a higher risk of mortality. This discovery is corroborated by studies conducted in Ethiopia [47] and Finland [53]. This phenomenon could be attributed to the increased mortality risk associated with neonates bearing additional congenital malformations, which consequently impacts the prognosis of CHD adversely.

While this study encompassed a large population and employed a follow-up approach, it encountered several limitations. Firstly, because of its retrospective design, certain maternal predictors like educational and nutritional status were not assessed. Secondly, managing missing values posed a challenge. Lastly, we did not track neonates discharged before 28 days of life, potentially leading to an underestimation of the incidence rate.

## Conclusion

The current research revealed higher mortality and incidence density rates compared to studies in developed nations. Additionally, factors such as sepsis, place of birth, maternal history of GDM, other congenital malformations, and type of CHD were identified as significant predictors for neonatal CHD mortality. To address this issue, collaboration between the Federal Ministry of Health, stakeholders, and the Ethiopian Cardiac Association is essential to enhance the quantity and quality of cardiac centers in the country. Moreover, healthcare providers should prioritize neonates with sepsis, additional congenital malformations, and cyanotic CHD. Lastly, reducing home deliveries can be achieved by expanding ANC service coverage.

### Supplementary Information

Supplementary Material 1.

Supplementary Material 2.

## Data Availability

The data set analyzed during the current study is available from the corresponding author upon reasonable request.

## Abbreviations

ANC: Antenatal Care
AHR: Adjusted Hazard Ratios
CHD: Congenital Heart Disease
CHR: Crude hazard ratio
GDM: Gestational Diabetes Mellitus
NICU: Neonatal intensive care units
USA: United States of America
VIF: Variance Inflation Factor
