# Supplementary material for: Incidence and predictors of mortality among neonates with congenital heart disease in Ethiopia: a retrospective cohort study
Source: BMC Pediatr. 2024 Aug 31;24:559. doi: 10.1186/s12887-024-05023-3 (PMC11365283; doi:10.1186/s12887-024-05023-3)
Supplement: Supplementary file 1 — Supplementary Material 1. [file 12887_2024_5023_MOESM1_ESM.docx]

**Annex 1: A life table showing survival probability of CHD in, Ethiopia, 2024**

| **Interval** | **Beg. Total** | **Deaths** | **censored** | **Survival** | **Std. Error** | **[95% Conf. Int.]** |
| --- | --- | --- | --- | --- | --- | --- |
| 1 2 | 583 | 2 | 19 | 0.9965 | 0.0025 | 0.9861- 0.9991 |
| 2 3 | 562 | 0 | 33 | 0.9965 | 0.0025 | 0.9861 -0.9991 |
| 3 4 | 529 | 3 | 31 | 0.9907 | 0.0041 | 0.9778 -0.9961 |
| 4 5 | 495 | 11 | 51 | 0.9675 | 0.0080 | 0.9474 -0.9800 |
| 5 6 | 433 | 4 | 43 | 0.9581 | 0.0092 | 0.9357 -0.9728 |
| 6 7 | 386 | 5 | 60 | 0.9446 | 0.0109 | 0.9188- 0.9624 |
| 7 8 | 321 | 6 | 42 | 0.9257 | 0.0131 | 0.8953- 0.9476 |
| 8 9 | 273 | 6 | 36 | 0.9039 | 0.0155 | 0.8685- 0.9302 |
| 9 10 | 231 | 2 | 28 | 0.8956 | 0.0165 | 0.8583- 0.9236 |
| 10 11 | 201 | 5 | 26 | 0.8718 | 0.0192 | 0.8288 -0.9046 |
| 11 12 | 170 | 3 | 29 | 0.8550 | 0.0211 | 0.8078- 0.8913 |
| 12 13 | 138 | 4 | 15 | 0.8288 | 0.0242 | 0.7752- 0.8707 |
| 13 14 | 119 | 1 | 20 | 0.8212 | 0.0251 | 0.7655- 0.8648 |
| 14 15 | 98 | 0 | 17 | 0.8212 | 0.0251 | 0.7655- 0.8648 |
| 15 16 | 81 | 3 | 8 | 0.7892 | 0.0302 | 0.7225- 0.8416 |
| 16 17 | 70 | 0 | 13 | 0.7892 | 0.0302 | 0.7225- 0.8416 |
| 17 18 | 57 | 1 | 5 | 0.7747 | 0.0329 | 0.7020 -0.8318 |
| 18 19 | 51 | 2 | 5 | 0.7587 | 0.0359 | 0.6795- 0.8209 |
| 19 20 | 45 | 0 | 3 | 0.7413 | 0.0391 | 0.6552 -0.8090 |
| 20 21 | 41 | 0 | 3 | 0.7413 | 0.0391 | 0.6552 -0.8090 |
| 21 22 | 38 | 0 | 4 | 0.7413 | 0.0391 | 0.6552- 0.8090 |
| 22 23 | 34 | 0 | 5 | 0.7413 | 0.0391 | 0.6552 -0.8090 |
| 23 24 | 29 | 0 | 8 | 0.7413 | 0.0391 | 0.6552- 0.8090 |
| 24 25 | 21 | 0 | 7 | 0.7413 | 0.0391 | 0.6552- 0.8090 |
| 25 26 | 14 | 0 | 4 | 0.7413 | 0.0391 | 0.6552- 0.8090 |
| 26 27 | 10 | 0 | 3 | 0.7413 | 0.0391 | 0.6552 -0.8090 |
| 27 28 | 7 | 0 | 3 | 0.7413 | 0.0391 | 0.6552- 0.8090 |
| 28 29 | 4 | 0 | 4 | 0.7413 | 0.0391 | 0.6552- 0.8090 |
