# Supplementary material for: Incidence and predictors of mortality among neonates with congenital heart disease in Ethiopia: a retrospective cohort study
Source: BMC Pediatr. 2024 Aug 31;24:559. doi: 10.1186/s12887-024-05023-3 (PMC11365283; doi:10.1186/s12887-024-05023-3)
Supplement: Supplementary file 2 — Supplementary Material 2. [file 12887_2024_5023_MOESM2_ESM.docx]

**DBCSH**

**1922**

**156**

**WCSH**

**1045**

**85**

**TGCSH**

**2677**

**218**

**GUCSH**

**1532**

**124**

**587**

| **LIST OF COMPREHENSIVE SPECIALIZED HOSPITALS** |
| --- |

| **GCSH** | **DBCSH** | **WCSH** | **FCSH** | **DMCSH** | **DTCSH** | **TGCSH** | **DCSH** |
| --- | --- | --- | --- | --- | --- | --- | --- |

| **BY SRS** |
| --- |

**(N=7176*)***

Total sample size

**Figure 1:** Schematic presentation of the sampling procedure for incidences and predictor of mortality among neonates with CHD in Ethiopia, 2024.

|       **Figure 2:** Kaplan meier graph for selected predictors of mortality to neoates with CHD in Ethiopia, 2024 |
| --- |

90.05%

9.949%

Censored

Died

**Figure 3:** Survival status of neonates with CHD in Ethiopia, 2024 (n=583)

**Figure 4:** Median survival time for neonates with CHD in Ethiopia, 2024

**Figure 5:** Cox-Snell residual cumulative hazard graph for neonates with CHD in Ethiopia, 2024
